# Mycobacteriophages as Incubators for Intein Dissemination and Evolution

**DOI:** 10.1128/mBio.01537-16

**Published:** 2016-10-04

**Authors:** Danielle S. Kelley, Christopher W. Lennon, SEA-PHAGES, Marlene Belfort, Olga Novikova

**Affiliations:** a Department of Biomedical Sciences, School of Public Health, University at Albany, State University of New York, Albany, New York, USA; b Department of Biological Sciences and RNA Institute, University at Albany, State University of New York, Albany, New York, USA; c Science Education Alliance Phage Hunters Advancing Genomics and Evolutionary Science Program (SEA-PHAGES), Howard Hughes Medical Institute, Chevy Chase, Maryland, USA

## Abstract

Inteins are self-splicing protein elements that are mobile at the DNA level and are sporadically distributed across microbial genomes. Inteins appear to be horizontally transferred, and it has been speculated that phages may play a role in intein distribution. Our attention turns to mycobacteriophages, which infect mycobacteria, where both phage and host harbor inteins. Using bioinformatics, mycobacteriophage genomes were mined for inteins. This study reveals that these mobile elements are present across multiple mycobacteriophage clusters and are pervasive in certain genes, like the large terminase subunit TerL and a RecB-like nuclease, with the majority of intein-containing genes being phage specific. Strikingly, despite this phage specificity, inteins localize to functional motifs shared with bacteria, such that intein-containing genes have similar roles, like hydrolase activity and nucleic acid binding, indicating a global commonality among intein-hosting proteins. Additionally, there are multiple insertion points within active centers, implying independent invasion events, with regulatory implications. Several phage inteins were shown to be splicing competent and to encode functional homing endonucleases, important for mobility. Further, bioinformatic analysis supports the potential for phages as facilitators of intein movement among mycobacteria and related genera. Analysis of catalytic intein residues finds the highly conserved penultimate histidine inconsistently maintained among mycobacteriophages. Biochemical characterization of a noncanonical phage intein shows that this residue influences precursor accumulation, suggesting that splicing has been tuned in phages to modulate generation of important proteins. Together, this work expands our understanding of phage-based intein dissemination and evolution and implies that phages provide a context for evolution of splicing-based regulation.

## IMPORTANCE

Inteins are mobile protein splicing elements found in critical genes across all domains of life. Mycobacterial inteins are of particular interest because of their occurrence in pathogenic species, such as *Mycobacterium tuberculosis* and *Mycobacterium leprae*, which harbor inteins in important proteins. We have discovered a similarity in activities of intein-containing proteins among mycobacteriophages and their intein-rich actinobacterial hosts, with implications for both posttranslational regulation by inteins and phages participating in horizontal intein transfer. Our demonstration of multiple insertion points within active centers of phage proteins implies independent invasion events, indicating the importance of intein maintenance at specific functional sites. The variable conservation of a catalytic splicing residue, leading to profoundly altered splicing rates, points to the regulatory potential of inteins and to mycobacteriophages playing a role in intein evolution. Collectively, these results suggest inteins as posttranslational regulators and mycobacteriophages as both vehicles for intein distribution and incubators for intein evolution.

## INTRODUCTION

Inteins are mobile protein splicing elements found in coding regions across genomes of many microbes. They possess the unique ability of self-catalyzed excision from a host precursor protein and ligation of the flanking polypeptides, termed exteins (1, 2). Inteins were discovered over 25 years ago in the vacuolar ATPase (*VMA1*) gene of *Saccharomyces cerevisiae* (3, 4). Sequence comparison to *Neurospora crassa* revealed that there was high homology with the exception of an internal portion of the protein (3, 4). It was eventually determined that this intervening spacer was an internal protein capable of excising itself from the hosting polypeptide (4). Since then, many inteins have been found through sequence-based approaches in a wide range of microbes, including bacteria, archaea, and some single-celled eukaryotes, as well as frequently in viral and bacteriophage genomes (5, 6). Interestingly, inteins appear to be absent from several notable bacterial model organisms and pathogens, including *Escherichia coli*, *Salmonella*, and *Vibrio cholerae* (5).

Within this broad distribution are several different types of inteins. Some are relatively short, carrying only the domains necessary for splicing. Other larger inteins have incorporated homing endonucleases (HENs), which are situated between the splicing domains. The HENs generally belong to the dodecapeptide family of endonucleases, characterized by the LAGLIDADG sequence (7, 8). Mobile inteins harness the power of HEN-mediated cleavage at a specific DNA sequence, termed the homing site, followed by gene conversion of an intein-free to an intein-containing allele. In return for its service, the HEN finds a “safe haven” within the protein splicing domains and avoids strong purifying selection associated with coding regions in streamlined microbial genomes.

Horizontal gene transfer appears to have played a role in the evolutionary history of inteins (9, 10). Although bacteriophages are well-known vectors for gene transfer, they remain largely unexplored for the presence and distribution of inteins. Bacteriophages, with an estimated 10^31^ bacterial and archaeal phage particles in the biosphere, comprise the majority of viral diversity. One of the key features in the evolution of the viral world is an extensive exchange of gene modules resulting in impressive diversity. Genome mosaicism is pervasive among bacteriophages, reflecting an unusually high degree of genetic exchange in their evolution (11, 12). Thus, elucidating the dynamics of bacteriophage inteins is instrumental for further advancing our understanding of intein evolutionary history.

Mycobacteriophages, a group of diverse, double-stranded DNA (dsDNA) bacteriophages, prey on mycobacteria, including *Mycobacterium smegmatis* and pathogenic *Mycobacterium tuberculosis* (13–15). Mycobacteria belong to the phylum *Actinobacteria*, which includes other notable members such as *Corynebacterium diphtheriae*, the causative agent of diphtheria, and *Streptomyces* species, sources of various antibiotics (16). The actinobacterial phylum is particularly intein rich, with over 48% of genomes containing inteins (5). The first bacterial intein was identified in the recombinase gene *recA* of *M. tuberculosis* by sequence comparison to *E. coli* (17, 18), followed by the discovery of another *recA* intein in the pathogen *Mycobacterium leprae* (19). Additional inteins have since been found among various mycobacterial species, often interrupting important genes like the replicative helicase *dnaB* and iron-sulfur scaffold *sufB* (6). As mycobacteriophages have been proposed to undergo frequent host expansion events (14), these phages provide an ideal background in which to investigate intein dynamics.

To learn how phages might contribute to intein evolution, we embarked on an intein search in mycobacteriophages, taking advantage of the ever-expanding repository of completely sequenced and often annotated genomes (13, 20). We find a wide variety of inteins across multiple mycobacteriophage groups, termed clusters, with many inteins localizing in important motifs of the host proteins. The majority of inteins are found in proteins specific to phages, such as the intein-rich large terminase subunit which is involved in generating the cohesive ends and DNA packaging into the procapsid, and in functional modules that are shared with their bacterial host. Several phage inteins are shown to be splicing competent and to encode active HENs for mobility. We find general evidence of intein flow and at least one clear example of horizontal intein transfer. Analysis of the intein sequences highlights differences between *Actinobacteria* and mycobacteriophages in conservation of a key catalytic residue, suggesting that phages select for intein features distinct from those in their bacterial hosts. We further demonstrate that this residue dramatically modulates splicing, which has important implications for both intein evolution and intein-based regulation and points to mycobacteriophages playing an important role in the evolutionary history of inteins.

## RESULTS

### Inteins are widely distributed among mycobacteriophages.

**FIG 1:**
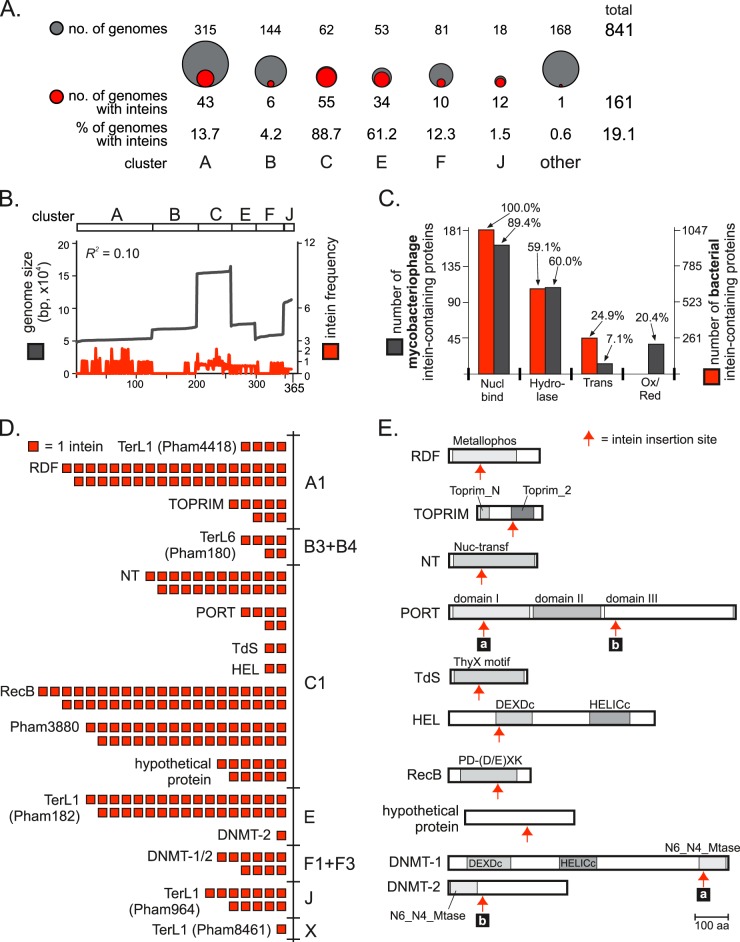
Overview of intein distribution in mycobacteriophages. (A) Distribution of inteins among mycobacteriophage clusters. The number of intein-positive genomes (red, value below the circle) was compared to the total number of sequenced phage genomes (gray, value above the circle) in a cluster. “Other” includes clusters D, G to I, K to Z, and singletons. (B) Distribution of inteins does not correlate with genome size. Vertical axis represents genome sizes (black) and frequency of inteins (red; number of inteins per 100 protein-coding sequences) in corresponding phages on the horizontal axis. Mycobacteriophage genomes (365 had protein-coding sequence numbers available) are organized by cluster. Coefficient of determination, *R*^2^ = 0.10. (C) Functional genomics of intein-containing proteins. Results for Gene Ontology (GO) term enrichment analysis of dominant functional categories of mycobacteriophage proteins with inteins are compared to those for bacterial intein-containing proteins. GO term enrichment of the 181 mycobacteriophages which gave GO terms (red) and 1,047 bacterial (gray) intein-containing proteins, previously analyzed (5). Dominant GO terms are shown: Nucl bind, nucleotide binding (GO:0003676); hydrolase (GO:0016787); Trans, transferase (GO:0016740); and Ox/Red, oxidoreductase (GO:0016491). The percentages of the associated proteins are indicated above the bars. (D) Intein distribution by host protein and phage clusters. Each square represents one intein. (E) An overview of intein-containing proteins indicates the intein insertion site relative to protein domains (arrow). Intein insertion sites for TerL and Pham3880 are shown in Fig. 2A. Abbreviations: TerL1, large terminase subunit terminase_1; TerL6, terminase_6; Pham3880, terminase-like; RDF, recombination directionality factor; TOPRIM, topoisomerase-primase; NT, DNA nucleotidyltransferase; PORT, portal protein; TdS, thymidylate synthase; HEL, helicase; RecB, RecB-like exonuclease; DNMT-1/2, DNA methyltransferase; Metallophos, metallophosphoesterase domain; Nuc-transf, nucleotidyltransferase domain; DEXDc and HELICc, domains associated with DEAD-like helicases; PD-(D/E)XK, nuclease domain; N6_N4_Mtase, DNA methylase; aa, amino acids.

A total of 841 mycobacteriophage genomes were surveyed, of which 161 (19.1%) were found to harbor inteins (Fig. 1A). The full list of analyzed genomes, phage clusters, and *in silico* search results for inteins is available in Tables S1 and S2 in the supplemental material. A total of 229 inteins were identified (Table 1), found sporadically distributed among mycobacteriophages across clusters, which provide a classification system for mycobacteriophages based on DNA sequence identity (21). While the number of available genomes in the database varies widely by cluster, we observed no relationship between heavily represented clusters and numbers of intein-containing phages (Fig. 1A). For example, in cluster C, 55 out of 62 genomes (88.7%) contain inteins, whereas in cluster B only 6 out of 145 genomes (4.2%) harbor inteins. Clusters also vary greatly in genome size (13). Whereas there is a strong correlation between genome size and number of protein-encoding sequences (*R*^2^ = 0.91), we observed no relationship between genome size and the frequency of inteins (*R*^2^ = 0.10) (Fig. 1B).

**TABLE 1:** Intein-containing proteins from mycobacteriophages

Protein	No. of inteins	POG^a^	Description/full name	Cluster distribution	Intein reference(s)
TerL1	50	POG0201	Phage terminase, large subunit	A1, E, J, X	12, 21, 36
TerL6	6	POG0042	Phage terminase, large subunit	B3, B4	
Pham3880	33	No hit	Terminase-like protein	C1	62
RecB	40	No hit	CRISPR-associated Cas4 RecB-like exonuclease	C1	21, 38
RDF	37	POG2995	Recombination directionality factor; putative calcineurin-like metallophosphoesterase (activity not shown)	A1	26
NT	23	POG1177	Nucleotidyltransferase	C1	20, 62
DNMT-1	7	POG0053	N6_N4_Mtase, DNA methylase N-4/N-6 domain-containing protein, methylation subunit methyltransferase	F1, F3	
DNMT-2	4	POG0990	N6_N4_Mtase, DNA methylase N-4/N-6 domain-containing protein	E, F1	
TOPRIM	8	POG1608	DNA primase/topoisomerase	A1	
PORT	6	POG1190	Portal protein	C1	62
HEL	2	No hit	Helicase, unknown function, related to HepA	C1	
TdS	2	POG1033	Flavin-dependent thymidylate synthase, ThyX-like protein	C1	62
Hypothetical protein	11	No hit	Hypothetical protein, unknown function	C1	62

a POG, Phage Orthologous Groups.

### Most mycobacteriophage inteins reside in nucleic acid binding proteins.

Next, we conducted functional genomic studies of mycobacteriophage inteins. To categorize mycobacteriophage intein-containing proteins, we utilized Phage Orthologous Groups (POGs) (Table 1) (22), in analogy to Cluster of Orthologous Groups, which was previously used to functionally classify exteins of bacterial intein-containing proteins (5). Inteins clustered in predominantly phage-specific proteins, including large terminase subunits (TerL), DNA methylases (DNMT-1/2), a putative topoisomerase-primase (TOPRIM), and portal proteins (PORT). To better compare the different complements of genes in phages and their hosts, we used Gene Ontology (GO) term enrichment (23) to analyze intein-containing data sets (Fig. 1C) (5). Strikingly, all phage and 84.9% of bacterial intein-containing proteins bind nucleic acid following splicing, of which ~60% possess hydrolase activity in both phage and bacteria. In contrast, differences are found in intein distribution in transferases, which are more common in the phage data set, and oxidoreductases, where inteins have been assigned only to bacterial proteins (5) and do not occur in mycobacteriophages.

### Intein enrichment in specific clusters and active centers of mycobacteriophage proteins.

**FIG 2:**
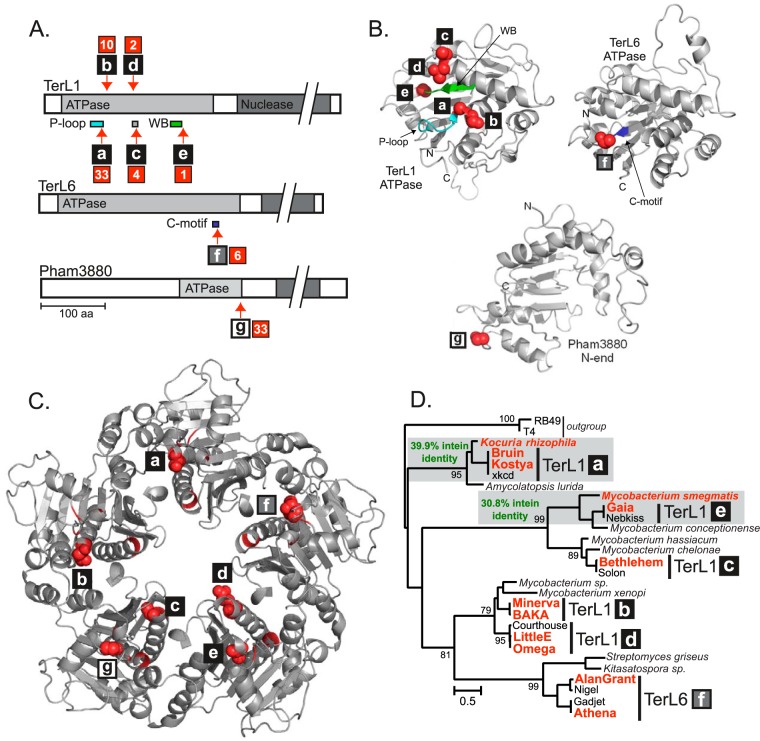
TerL inteins concentrate in the ATPase domain near functional motifs. (A) Overview of intein insertion sites among three types of terminase-like proteins show that all localize to the ATPase domain. TerL1 has five unique intein insertions, a to e**,** while TerL6 proteins and Pham3880 each have single insertions, f and g, respectively (red arrows). Values in red squares indicate the number of inteins at each site. P-loop, cyan; Walker B motif (WB), green; C-motif, blue. (B) ATPase structure models of three terminase-like proteins. Insertion sites are shown as red spheres, and motif coloring corresponds to panel A. Models are represented as follows: TerL1, Minerva gp9 (residues 1 to 242); TerL6, Chandler gp6 (residues 59 to 309); Pham3880, ScottMcG gp245 (residues 107 to 317). Full structure models are in Fig. S1 in the supplemental material. (C) Intein insertions mapped onto a TerL pentamer structure. The intein insertion sites were mapped on a solved TerL ATPase domain structure from the virus P74-26 (PDB 4ZNL) (34). Intein insertions are shown once at each site as red spheres and indicated in red on the other monomers. (D) TerL phylogenetic tree. Maximum-likelihood (ML) tree for intein-containing and related intein-free mycobacteriophage and actinobacterial prophage TerLs was constructed. Intein-containing phages and actinobacterial prophages from *K. rhizophila* and *M. smegmatis* are indicated in red. Gray shading indicates the bacterial prophage and mycobacteriophage inteins that were compared by protein pairwise alignment, with the percent identity indicated (green). Values for significant external nodes higher than 75% are shown. T4 gp17 and RB49 gp17 are used as an outgroup. Scale indicates the number of substitutions per site. Mycobacteriophage TerL intein insertions (a to f) are indicated.

We classified intein-containing proteins into groups based on their sequence and structural similarity to proteins of known function. Thirteen distinct groups of mycobacteriophage proteins showed the presence of inteins (Table 1; also see Table S2 in the supplemental material). The majority of inteins, in terms of both number and diversity, are found in subcluster C1 (Fig. 1A and D), with seven unique intein-containing genes and six inteins exclusive to this subcluster. While some inteins are confined to a single cluster, others are present across multiple clusters and subclusters (Fig. 1D; see also Table S2). Additionally, we observed that the viral DNA packaging protein TerL is the most abundant intein-containing protein (~40% of all mycobacteriophage inteins) (Fig. 1D; Table 1) and is considered in detail below (Fig. 2).

Besides TerL, we identified another relatively large group of intein-containing proteins as a putative clustered regularly interspaced short palindromic repeat (CRISPR) Cas4-like exonuclease belonging to the RecB-like family of proteins. Although they were originally described as HNH endonucleases in the Actinobacteriophage database, we could not detect the conserved HNH motif (24). Motif searches and structural modeling indicated a family of CRISPR-associated Cas4 RecB-like exonucleases as a more appropriate placement (25), although these proteins may not be CRISPR related functionally. In total, 40 intein-containing RecB-like proteins were found among C1 mycobacteriophages, representing the second largest group of intein-containing proteins (Fig. 1D; Table 1). A single intein insertion point is located in one of the highly conserved motifs of the nuclease, PD-(D/E)XK (Fig. 1E).

Insertions next to conserved motifs, often after invariant amino acid residues, are a theme that extends to other inteins (Fig. 1E) (5). Mycobacteriophage recombination directionality factors (RDFs) carry inteins next to an absolutely conserved motif within the metallophosphoesterase domain (26), whereas the intein insertion point in TOPRIM localizes to the active site (Fig. 1E). This observation extends to the rest of the mycobacteriophage intein-containing proteins, such as nucleotidyltransferase-like protein (NT; 23 examples), thymidylate synthase (TdS; 2 examples), hypothetical helicase (HEL; 2 examples), and two families of putative DNMT-1/2 (11 inteins total). All these proteins are involved in either DNA modification or nucleotide metabolism.

### Terminase-like proteins are the primary intein-containing sequences in mycobacteriophages.

The most abundant and diverse group of phage inteins was found in TerL and terminase-like proteins (Fig. 1D and 2; Table 1). TerL is part of a hetero-oligomeric complex together with the small terminase subunit, which cleaves the concatemeric phage DNA to generate the cohesive ends and packages the mature DNA into the procapsid during lytic growth (27). TerL belongs to the P-loop-containing nucleoside triphosphate (NTP) hydrolase superfamily, all members of which are AAA ATPases (28). There are at least four diverse AAA-like terminase families (29, 30), but only terminase_1 (TerL1) and terminase_6-like (TerL6) proteins have inteins. TerL1 proteins are the largest group, with 50 intein-containing representatives across four mycobacteriophage clusters/subclusters and protein phamilies (Pham), followed by inteins in TerL6 proteins, present in B3 and B4 phages (Fig. 1D and 2). A terminase-like protein in C1 phages was also found to have inteins and was annotated by the protein phamily Pham3880 (20, 31).

An individual large subunit of the terminase complex is comprised of an N-terminal ATPase and C-terminal nuclease (27). Strikingly, terminase inteins localize to the N-terminal ATPase domain, with seven unique insertion sites (Fig. 2A), designated by lowercase letters following the protein name, e.g., TerL1-a (6, 8). To better appreciate the distribution of the inteins relative to key motifs and structural features of the ATPase domain, structures were predicted using homology modeling (Fig. 2B; also see Fig. S1 in the supplemental material). The most common TerL1 intein insertions, a and b, are 1 amino acid residue apart and located in the P-loop Walker A motif involved in ATP binding (Fig. 2A and B) (27). The TerL1-a intein is inserted between the invariant Lys and nucleophilic Thr in a “classic” P-loop intein insertion, common among bacterial and archaeal intein-containing proteins (5, 32, 33). TerL1-c, -d, and -e inteins are less frequent, inserted in either a poorly conserved helix of unknown function (TerL1-c and -d) or the Walker B (WB) motif (TerL1-e) (Fig. 2A) (27). TerL6-f inteins are found in a putative ATPase coupling motif, or C-motif (Fig. 2A and B) (30). Finally, 33 inteins inserted at the C-terminal end of the ATPase domain were found in the TerL-like Pham3880 protein. Pham3880 has only 75 amino acid residues of the ATPase domain and is missing motifs such as the P-loop. However, the model resembles that of TerL, and the intein insertion point was designated Pham3880-g (Fig. 2A and B; also see Fig. S1).

In addition to being part of a hetero-oligomeric complex, TerL forms a homopentamer (34). As many inteins are found in proteins that make higher-order complexes (5), we asked how the TerL inteins fit in this context. Mapping of the insertion sites on the TerL pentamer shows that higher-order complexes are unlikely to form with an intein present (Fig. 2C), making splicing a crucial step in generating the active site and in complex formation.

Many prophages have TerL genes with inteins, including in *Actinobacteria* (5), and we wanted to understand how these prophage TerLs relate to our intein-containing mycobacteriophages. Therefore, phylogenetic analysis based on TerL sequences from mycobacteriophages and related prophages was performed, showing well-defined groups among the phage terminases (Fig. 2D). The intein-containing TerLs in *M. smegmatis* and *Kocuria rhizophila* prophages cluster with TerL1-e and TerL1-a, respectively (Fig. 2D). Analysis of the intein sequences showed that the TerL1-e intein has a 30.8% overall amino acid identity to the *M. smegmatis* prophage intein (Fig. 2D), in line with general intein relatedness of ≤30% reported previously (8) and observed during our analysis (see Fig. S2A in the supplemental material). However, the TerL1-a inteins have a 39.9% amino acid identity with the *K. rhizophila* TerL intein (Fig. 2D), higher than expected for typical intein resemblance. Notably, *K. rhizophila*, a member of the *Micrococcaceae* family, is not considered a host for mycobacteriophages, providing a potential example of extended host range. These intein-containing prophages are likely intermediates of intein transfer into bacterial genomes.

### Terminase inteins are splicing competent and endonuclease active.

**FIG 3:**
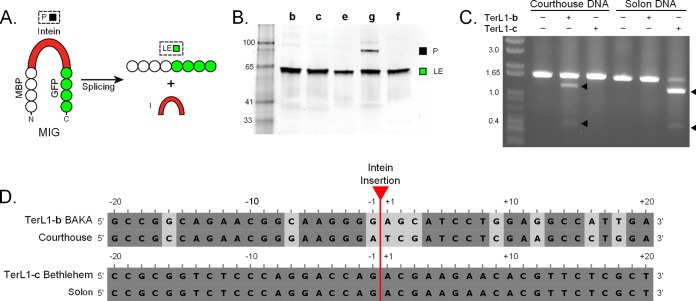
TerL inteins are splicing competent and can have active endonucleases. (A) MIG reporter system. The intein of interest was cloned between maltose binding protein (MBP) and GFP. Precursor (P) and ligated exteins (LE) are visualized by in-gel fluorescence. (B) TerL inteins are able to splice. Five representative TerL inteins cloned into the MIG reporter were assayed for splicing. Inteins are indicated by the insertion site letter. All five inteins investigated spliced quickly, primarily resulting in LE. Representative inteins are as follows: TerL1-b, BAKA gp6; TerL1-c, Bethlehem gp10; TerL1-e, Gaia gp2; TerL6-f, Chandler gp6; Pham3880-g, ScottMcG gp245. The numbers indicate the marker size in kDa. (C) TerL inteins have endonuclease activity. BAKA TerL1-b and Bethlehem TerL1-c inteins were tested for endonuclease activity against an inteinless TerL sequence from related phages, Courthouse and Solon, respectively. Cleavage products (black arrowheads) for both are ~1.3 kb and 0.4 kb. The DNA substrate was mixed with buffer, lysate with overexpressed unrelated TerL intein, or lysate with overexpressed related TerL intein. The numbers indicate the marker size in kb. (D) Sequence identity at TerL intein insertion sites. Sequence flanking the TerL intein insertion site (20 nucleotides up- and downstream) for each phage pair was analyzed, with high sequence identity among pairs (BAKA-Courthouse, 75%; Bethlehem-Solon, 100%). Conservation is shown by shades of gray. Data are representative of at least three independent experiments.

To gain insight into the enzymatic functions of the highly represented TerL inteins, protein splicing and endonuclease cleavage assays were performed. Five of the seven TerL inteins were tested for splicing by cloning the intein genes, plus 7 to 10 native flanking residues, into a MIG (maltose binding protein [MBP]-intein-green fluorescent protein [GFP]) fusion construct. The MIG system allows monitoring of splicing activity by in-gel fluorescence, where GFP-containing products are visible (Fig. 3A) (35). All inteins spliced readily, and ligated extein (LE) was the predominant product for all terminase inteins, with precursor (P) readily visible for only Pham3880-g (Fig. 3B).

HEN-based cleavage of intein-less alleles is another important aspect of intein function, vital for intein mobility and invasion of novel niches. To ascertain cleavage activity of the TerL inteins, we selected two phage pairs, with an intein-containing phage and its inteinless partner. Thus, BAKA TerL1-b and Bethlehem TerL1-c inteins were tested for endonuclease activity against inteinless TerL sequences in closely related partner phages, Courthouse and Solon, respectively (Fig. 3C). These partner phages belong to the same cluster, with general conservation of sequence around the insertion sites (Fig. 3D). Lysate containing intein and target sequence was incubated, and cleavage was observed with both inteins (Fig. 3C). Further, the activity was specific for the partner target TerL gene, with the TerL1-b intein from BAKA active against Courthouse but not Solon DNA and vice versa. The observed activity corresponded with the presence of an identifiable HEN domain in TerL1-b and TerL1-c (see Fig. S3 in the supplemental material).

### Putative horizontal transfer of inteins.

**FIG 4:**
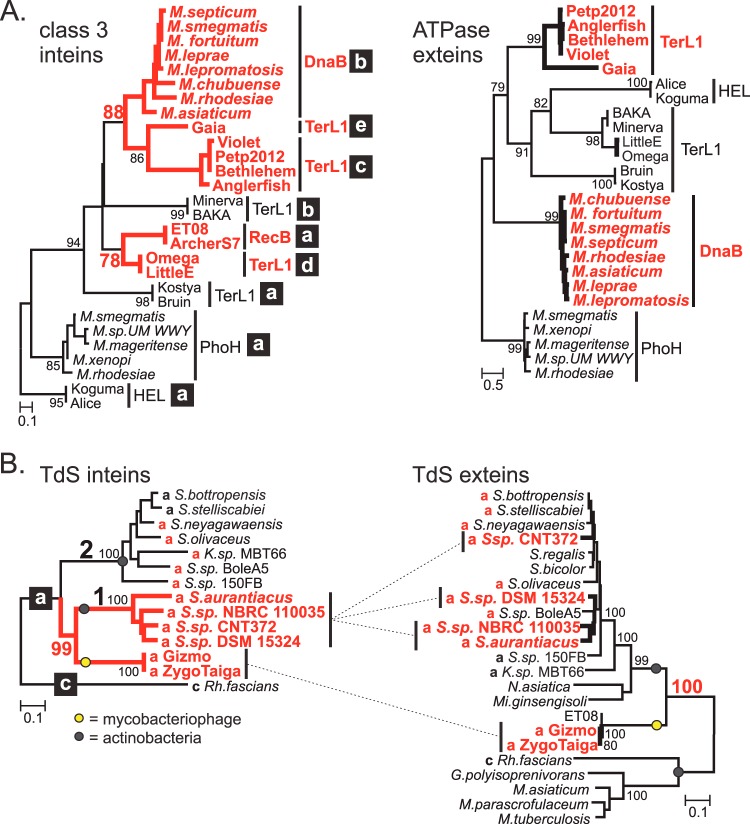
Putative horizontal transfer of inteins. (A) Evidence of common ancestry among phage and mycobacterial inteins. Phylogenetic analysis (ML) of class 3 mycobacteriophage/mycobacterial inteins (left) and their ATPase-containing exteins (right), excluding RecB. The intein tree shows two examples of supported clustering (red), including mycobacteriophage TerL1-c/e and mycobacterial DnaB-b inteins, indicating a common ancestor. The exteins group independently from their inteins. Inteins were aligned based on splicing blocks; exteins were aligned based on the ATPase domain. Full trees for class 1 and 3 inteins are in Fig. S2 in the supplemental material. (B) Putative horizontal transfer of TdS inteins. Phylogenetic analyses of TdS inteins (left) and TdS proteins (right), some with inteins. Incongruence in clustering of the two trees implies horizontal intein transfer (red). The presence of an intein is indicated by its insertion site a or c. For both panels, trees are unrooted and values for significant external nodes higher than 75% are shown. Scale indicates the number of substitutions per site. Genus abbreviations are as follows: *M*, *Mycobacterium*; *S*, *Streptomyces*; *K*, *Kitasatospora*; *Rh*, *Rhodococcus*; *N*, *Nocardia*; *Mi*, *Microbacterium*; *G*, *Gordonia*.

To better establish how mycobacteriophage and mycobacterial inteins are related, phylogenetic trees were generated. As there is high conservation in the intein sequence from the same insertion site group, often 100% identity, the analysis was performed with two representative mycobacteriophages for each insertion group. Whereas class 1 inteins showed no cases of putative phage-mycobacterial transfer (see Fig. S2B in the supplemental material), there are two groups in class 3 inteins which are suggestive. First, phage inteins in RecB and TerL1-d clustered together with relatively high statistical support and have 48.0% identity, implying a recent common ancestor (Fig. 4A). Second, mycobacterial DnaB-b and mycobacteriophage TerL1-c and -e inteins form a common clade. In contrast, analysis of DnaB and TerL1 exteins (ATPase domain) does not reveal a similar clade (Fig. 4A). As the DnaB-b inteins lack endonucleases, the splicing domains were used for pairwise sequence analysis. Excitingly, the mycobacteriophage inteins have a high percentage of amino acid sequence identity with those of mycobacterial DnaB-b inteins, ranging from 41.1% to 51.6% for TerL1-c and 52.6% to 54.7% for TerL1-e (see Table S3), which strongly suggests intein transfer between phage and host.

Whereas the majority of inteins described here share less than 30% identity between phage and host (see Fig. S2A in the supplemental material), except as noted above (Fig. 2D and 4A), a high degree of identity (48.4%) is also found between the TdS inteins of mycobacteriophages and the inteins from certain actinobacterial *Streptomyces* species (Fig. 4B). To further probe the relationship of TdS and its inteins, we reconstructed a TdS intein-based phylogenetic tree (Fig. 4B, left) and a corresponding extein-based tree, including TdS proteins lacking inteins (Fig. 4B, right). A closer look shows that among *Streptomyces* and close relatives, there are two TdS intein clades at the same TdS-a insertion point, designated groups 1 and 2 (Fig. 4B, left). While group 2 TdS inteins are widely distributed among *Streptomyces* species (Fig. 4B; only a few examples of TdS intein-containing *Streptomyces* are shown), only four group 1 TdS *Streptomyces* inteins are identified, shown in red. As seen from the TdS intein phylogeny, group 1 TdS inteins are more closely related to TdS inteins from mycobacteriophages Gizmo and ZygoTaiga than to group 2. However, this is not the case for the exteins, as all TdS proteins from *Streptomyces* group together regardless of the corresponding TdS intein group (Fig. 4B, right), indicating recent common ancestry for exteins. The clustering discrepancy between the group 1 TdS inteins and their exteins (dashed line) indicates independent acquisition of group 1 and group 2 TdS inteins and implies horizontal intein transfer among mycobacteriophages and *Streptomyces*.

### Splicing modulation in noncanonical phage inteins.

**FIG 5:**
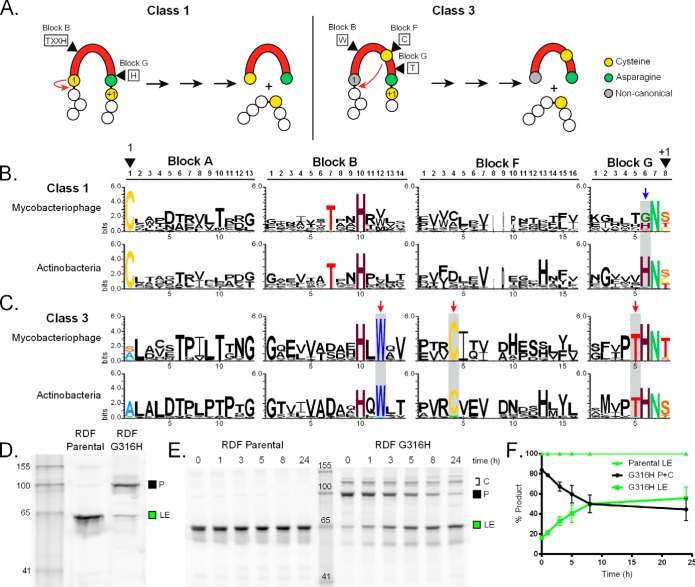
Lack of penultimate residue conservation among mycobacteriophages is modulatory. (A) Differences between class 1 and 3 inteins. Residues of interest in the splicing blocks for each class are boxed. Class 1 inteins initiate splicing using the first cysteine (1; yellow), which acts as a nucleophile and attacks the preceding amide bond (red arrow). In contrast, class 3 inteins use an internal cysteine in block F (yellow) to initiate splicing (red arrow). Both pathways then proceed to completion (black arrows), resulting in excised intein and ligated exteins. The full mechanism for both classes can be found in Fig. S4 in the supplemental material. (B) Disparity of class 1 intein residue. Logos for class 1 blocks of mycobacteriophage and actinobacterial inteins show key residues (colored). The 1 (block A1) and +1 (block G8) residues are marked. The variation of the penultimate His (block G6) is highlighted (blue arrow; shading). (C) Conservation of class 3 intein residues. Comparison between phage and bacterial sequence logos is similar to that in panel B. The class 3 WCT triplet is indicated by red arrows and shading. (D) Mutation of the RDF penultimate residue to His leads to precursor accumulation. The Bethlehem gp51 RDF intein, with an R157W endonuclease-inactivating mutation, was cloned into the MIG reporter construct (RDF Parental) and the penultimate Gly mutated to the canonical His (RDF G316H). Splicing levels were compared, showing a dramatic increase in P accumulation with the G316H mutant relative to Parental. The numbers indicate the marker size in kDa. (E) Splicing of MIG RDF Parental and G316H over time. MIG RDF Parental and G316H lysates were allowed to splice over time. While RDF Parental has faint visible P, it is primarily processed to LE by time zero. In contrast, the RDF G316H mutant is able to slowly splice over time. There are also higher bands that correspond to disulfide-bonded precursor conformers (C). The numbers indicate the marker size in kDa. (F) Quantitation of MIG RDF splicing. The splicing of RDF parental and G316H over time was quantitated, and the ratios of P+C and LE were plotted. The faint P band visible for RDF Parental was not above background during quantitation. Data are representative of at least three independent experiments.

Inteins have conserved sequence blocks (A, B, F, and G) that contain the residues necessary for splicing. Blocks A and B comprise the N-terminal splicing domain, while blocks F and G make up the C-terminal splicing region (7, 8). Specific amino acids within these blocks are indicative of the intein class and splicing mechanism. There are three known splicing classes: class 1, the canonical pathway, and classes 2 and 3, which use alternative mechanisms (Fig. 5A; see also Fig. S4 in the supplemental material) (1, 36, 37). Our analysis indicates that mycobacteriophage inteins are of classes 1 and 3. Class 1 inteins use the nucleophile at position 1 to initiate splicing (Fig. 5A, class 1), while class 3 inteins, identifiable by the presence of a conserved Trp-Cys-Thr (WCT) triplet motif, have the initial nucleophilic attack performed by an internal cysteine (Fig. 5A, class 3) (36, 38).

The four splicing blocks of the mycobacteriophage inteins were identified (see Fig. S5 in the supplemental material) and subsequently compared to actinobacterial inteins from InBase (6), allowing the generation of sequence logos (Fig. 5B and C). We find conservation of catalytic residues among the members of class 1 between phages and *Actinobacteria*, with the notable exception of the penultimate His, which is typically a conserved intein residue (Fig. 5B) (8). This block G His, which assists terminal Asn cyclization (39), is poorly conserved among mycobacteriophage inteins, being replaced in some cases by Gly, Lys, and Ser (Fig. 5B, blue arrow). In sharp contrast, actinobacterial inteins strongly conserve His at this position.

The class 3 inteins lack nucleophiles at position A1 and are characterized by the WCT triplet in the B, F, and G blocks at positions 12, 4, and 5, respectively (see Fig. S5 in the supplemental material). We find that the WCT triplet is highly conserved among both phage and actinobacterial class 3 inteins (Fig. 5C, red arrows). We also note that all investigated inteins have Cys as the initiating nucleophile (Fig. 5B and C; see also Fig. S5).

To further investigate the penultimate His divergence among class 1 phage inteins, we compared splicing of such a phage intein to a mutant version with the canonical His. The intein from RDF, which has a penultimate Gly plus several flanking native residues, was cloned into the MIG reporter system (Fig. 3A) (35). Due to endonuclease-related toxicity, we recovered an endonuclease-inactivating mutant (R157W) in the presumptive DNA-binding region that does not impact splicing for subsequent mutagenesis. The parental intein splices completely, with ligated exteins being the primary product, whereas the G316H mutant has greatly increased amounts of precursor with only a trace of ligated exteins (Fig. 5D). A time course experiment indicates that while the parental MIG construct is again completely spliced at time zero (100% LE), the G316H mutant takes >5 h for splicing to be ~50% complete (Fig. 5E and F). A high-molecular-weight band (C) is also observed with the mutant and appears to be a precursor conformer resulting from intramolecular disulfide bonding, as the band disappears after treatment with reducing agents tris(2-carboxyethyl)phosphine (TCEP) or dithiothreitol (DTT) (data not shown).

## DISCUSSION

Here, we focus on phages that infect mycobacteria, providing the first comprehensive look at intein distribution, localization, and the relationship of these inteins to their bacterial counterparts. These inteins were identified by mining the plethora of available genomes curated in the Actinobacteriophage database. We present evidence of mycobacteriophages participating in intein dissemination among both phages and bacteria, show a global commonality in the types of proteins that host inteins, and advocate that phages allow for intein evolution. These studies inform a narrative on the potential role of phages in intein dissemination, how phages may facilitate intein evolution, and in turn, how inteins might have adapted to phage function.

Genes encoding proteins involved in replication, recombination, and repair are routinely found in phages (40), and we previously demonstrated that many such proteins have intein insertions in bacteria (5). GO term enrichment analysis indicates a striking global commonality in activities and functions of distinct intein-containing proteins across phages and bacteria, specifically nucleic acid binding and hydrolase, including ATPase, activities (Fig. 1C). The majority of mycobacteriophage inteins localize to functional motifs (Fig. 1E and 2), in line with prior observations (5).

Further, previous analysis of bacteria and archaea has shown that inteins have a propensity for P-loop ATPases, with ~70% of inteins found in ATP binding proteins (5, 32), and we find a similar penchant of mycobacteriophage inteins for ATPases, exemplified by TerL (Fig. 2). Not only are there seven independent insertions in distinct TerL proteins across multiple phage clusters (Fig. 1D and 2A), but the inteins all localize to the ATPase domain (Fig. 2), indicating a selection for inteins in TerL ATPases. The efficient splicing of the TerL inteins (Fig. 3B) and intein insertions in the context of the larger TerL complex suggests that splicing is necessary for function (Fig. 2C). This biased localization has been described as indicating selective retention and a potential role for inteins as modulators of expression of their host protein by acting as environmental sensors (5). Indeed, biochemical evidence supporting the regulatory capacity of inteins has begun to accumulate (33, 35, 41, 42). Several mechanisms have been described, including modulation by cysteine chemistry (35, 41), which is intriguing as Cys functions as the exclusive initiating nucleophile in mycobacteriophage inteins (Fig. 5B and C; see also Fig. S5 in the supplemental material). Our findings that mycobacteriophage inteins localize to similar sites in distinct phage-specific proteins and insert into important functional motifs further advocate the idea of a functional role for certain inteins.

An exciting aspect of intein dynamics, to date largely unaddressed, is their potential for horizontal transfer between genomes. Infiltration of a novel niche may involve exposure to a mobile intein, but the gene transfer vectors remain speculative (10, 43). As mycobacteria are not naturally competent, mycobacteriophages and conjugation are thought to function as the primary mechanisms of gene acquisition (44). It is conceivable that bacteriophages function as vectors for horizontal intein transfer, accidentally picking up intein sequences from host genomes during replication (45, 46) or invasion through HEN-mediated mobility and, reciprocally, depositing inteins in bacterial genomes. The demonstration of active mycobacteriophage intein endonucleases strengthens the argument for targeted intein invasion (Fig. 3C).

To address questions of horizontal transfer, we compared inteins within mycobacteriophages, between these phages and mycobacteria, and to bacteria in general. Evidence of intein dissemination among mycobacteriophages is apparent, with several intein groups present across multiple clusters and subclusters (Fig. 1; Table 1). Dissecting the cause of this distribution is challenging, as mycobacteriophages are known to be mosaic, with recombination often resulting in exchange of DNA (12). However, the abundance of inteins in TerL is less likely to be due to general recombination, as the exteins are distinct proteins (Fig. 1D; Table 1), and there appear to be multiple intein insertions within a confined genetic space, arguing in favor of homing-based invasion. Why certain clusters of mycobacteriophages are intein rich relative to others is unclear. Possible explanations are that the distinct and divergent histories of mycobacteriophage clusters (13, 14) have resulted in differential exposure and acquisition of inteins or that retention of inteins in response to specific selection pressure leads to the propagation of these mobile elements among certain clusters and not others.

To explore transfer between mycobacteriophages and bacteria, we analyzed intein-containing mycobacteriophage proteins compared to bacterial proteins (5). Phylogenetic comparison of inteins suggests that some bacterial and phage inteins have common ancestry, such as the class 3 DnaB-b and TerL1-c and -e inteins (Fig. 4A). This is further supported by the high percent identity between the splicing domains of these inteins, up to ~55% for TerL1-e to several mycobacterial species, including *M. smegmatis* (see Table S3 in the supplemental material). As phages have been proposed to be the origin for class 3 inteins (38), this supports a role for phages in intein spread, with transfer accounted for by site variation tolerance of the HEN (47).

Mycobacteriophages have also been implicated in the diversification of their hosts, which are known to contain prophages and prophage-like elements (48). Prophages can function as an intermediate step of horizontal transfer, having integrated into the host genome, which can provide more opportunities for intein movement. Phylogenetic analysis shows two examples of clustering of intein-containing terminase proteins of prophages in *K. rhizophila* and *M. smegmatis* with intein-containing terminases in mycobacteriophages, TerL1-a and -e, respectively (Fig. 2D). While the *M. smegmatis* intein is only somewhat similar to the TerL1-e intein, the intein from nonnative mycobacteriophage host *K. rhizophila* shares a high percent identity (39.9%) to the TerL1-a inteins (Fig. 2D), pointing to the potential for prophages as a gateway for widespread intein movement into bacterial genomes.

A more robust candidate for horizontal transfer is the intein present in TdS, a protein known to be horizontally transferred (49). The presence of related TdS inteins in mycobacteriophages and *Streptomyces* with disparately related exteins strongly points to horizontal movement of the intein (Fig. 4B). Interestingly, this intein belongs to the highly intein-rich C1 mycobacteriophage subcluster (Fig. 1A and D), which has been proposed to be relatively new to mycobacteria (20). While horizontal transfer is apparent for the TdS intein, we lack data to suggest a specific direction or nature of this transfer. Independent intein acquisition by C1 mycobacteriophages and *Streptomyces* and involvement of a third party are a possibility. Regardless of the directionality of movement, we provide compelling evidence of horizontal transfer of inteins between phages and bacteria.

The presence of inteins in bacteriophages and other viruses adds another level of complexity to the evolutionary dynamics of inteins. In general, double-stranded DNA (dsDNA) bacteriophages have higher mutation rates than bacteria (50) and diversification of inteins can be expected. Indeed, comparative analysis revealed interesting intein variants underrepresented in bacteria, including class 1 inteins lacking the highly conserved penultimate His (Fig. 5B). This His plays an important role in splicing, facilitating terminal Asn cyclization (see Fig. S4, class 1, step 3, in the supplemental material) (39). Our results with the RDF intein, which has Gly at the penultimate position, show that splicing is dramatically slowed when Gly is replaced with His (Fig. 5D to F). This result should be viewed in the context of previous studies of inteins with noncanonical penultimate residues in archaea and chloroplasts that have shown disparate responses when mutated. Some of these unusual inteins have increased splicing when His replaces the native penultimate residue (51, 52), some become splicing impaired (51), and others have no detectible change (53).

The penultimate residue may be one that is subject to selection because the role of this His can be assumed by an upstream His in block F (51, 53). RDFs control the directionality of integrase-dependent site-specific recombination and, in mycobacteriophages, are atypical, binding directly to integrase rather than DNA to exert function, and they have additional roles during lytic growth, likely in DNA replication (26, 54). The loss of His at this position may be an advantageous adaption by the phages to more quickly generate functional protein under normal conditions, and many of the other inteins with alternative penultimate residues are in proteins with DNA metabolism and replication functions (Table 1; also see Fig. S5 in the supplemental material). The increased propensity for variation at the penultimate position points to phages providing a space for evolution as inteins sample alternative catalytic residues that change the splicing rate, thereby regulating the host protein function.

The prevalence of inteins across kingdoms combined with mounting evidence that inteins may function as posttranslational regulators points to a need to understand where such mechanisms developed and how inteins have become so widespread. Our data that support horizontal intein transfer as well as selection of noncanonical catalytic residues that modulate splicing suggest that mycobacteriophages have participated in both the dissemination and the evolution of inteins.

## MATERIALS AND METHODS

### Intein survey from mycobacteriophage genomes.

Mycobacteriophage genomic sequences utilized in this study are available at the Actinobacteriophage database (http://phagesdb.org) and the genome database of the National Center for Biotechnology Information (NCBI; http://www.ncbi.nlm.nih.gov/genome). The source of individual genomes and other relevant data are listed in Table S1 in the supplemental material. All downloads were performed before 20 May 2015; 841 genomic sequences were accessible at that time (see Table S1). The primary search for intein-like sequences was performed using HMMER3 tools implemented in Unipro UGENE (v1.16.2; http://ugene.net) (55). We used two HMM 3 profiles constructed based on a multiple alignment of either the protein splicing domain sequences or the HEN sequences. An additional check for the presence of protein splicing domains and HEN domains was performed by BLAST analysis (http://blast.ncbi.nlm.nih.gov/Blast.cgi). Classification of detected inteins was performed based on the identity of their putative extein sequences and insertion sites, which are conventional approaches in intein classification (6, 8). We identified the putative open reading frame (ORF) encoding both extein and intein using the ORF Find feature in Unipro UGENE. The NCBI BLASTp (http://blast.ncbi.nlm.nih.gov/Blast.cgi) and local BLASTp against Actinobacteriophage databases (http://phagesdb.org/blastp) were then used to identify similarity with already-annotated proteins. Final annotation was achieved when possible using the NCBI Conserved Domain Database search service (CD Search; http://www.ncbi.nlm.nih.gov/Structure/cdd/wrpsb.cgi). The intein database, InBase (6), was used for cross-referencing.

### Further sequence and phylogenetic analysis and modeling.

Multiple sequence alignments of amino acid and nucleotide sequences were done in Unipro UGENE. For amino acids, the MUSCLE alignment package was used (56), and intein percent identity was based on comparison of the full intein sequence, unless otherwise noted. For DNA alignments, a pairwise Kalign algorithm was implemented (57). All phylogenetic trees were generated using the maximum-likelihood (ML) method in PhyML (58) (http://www.phylogeny.fr). A nonparametric Shimodaira-Hasegawa-like approximate likelihood-ratio test (SH-aLRT) was used to evaluate statistical support (59). Logos for the sequence blocks were generated from alignments using WebLogo3 (http://weblogo.threeplusone.com). The InterPro database was used in GO enrichment analysis (http://www.ebi.ac.uk/interpro/).

Structure models of representative terminase proteins were generated by Phyre2 servers (60). TerL1 (Minerva gp9) was modeled with a coverage of 444 residues (87%), TerL6 (Chandler gp6) was modeled with a coverage of 432 residues (73%), and Pham3880 (ScottMcG gp245) was modeled with a coverage of 376 residues (51%). Model coverage was at a confidence level of >90% accuracy. The five TerL1 insertions were mapped onto a single model based on the ATPase alignment described above. The pentameric TerL ATPase domain complex structure from P74-26 (PDB 4ZNL) was kindly provided by Brian Kelch (34). The mycobacteriophage intein insertion sites in the P74-26 sequence were determined by a secondary structure alignment with PROMALS3D (61). Models and structures were manipulated in PyMOL (v1.7.2), and +1 intein residues and important functional motifs are indicated.

### MIG cloning, MIG splicing assays, and cleavage assays.

MBP-intein-GFP (MIG) reporter constructs were made to monitor splicing of RDF and TerL inteins, as previously described (35). Plasmids and strains are listed in Table S4 in the supplemental material. Briefly, the RDF intein from Bethlehem and TerL inteins from five mycobacteriophages (BAKA, Bethlehem, Gaia, ScottMcG, and Chandler), plus 7 to 10 native residues (see Table S4), were amplified from mycobacteriophage lysates, kindly donated by Graham Hatfull, using Q5 High-Fidelity DNA polymerase (NEB) for the RDF intein and CloneAmp HiFi PCR Premix (Clontech) for TerL inteins. Oligonucleotides from IDT (Integrated DNA Technologies) are listed in Table S5 in the supplemental material. The vector, pACYC-Duet with the MIG cassette, was linearized with SphI and ClaI (NEB). DNA fragments were visualized by electrophoresis in 1% agarose gels using EZ-Vision DNA dye (Amresco), excised, and purified using the Zymoclean gel DNA recovery kit (Zymo Research). Intein fragments were cloned at the SphI/ClaI sites, between MBP and superfolder GFP coding sequences, using the In-Fusion HD Cloning Plus kit (Clontech). Plasmid DNA was isolated using the QIAprep Spin Miniprep kit (Qiagen), and clones were verified by sequencing (Eton Bioscience). For MIG RDF, the G316H mutant was made using the QuikChange Lightning Multi site-directed mutagenesis kit (Agilent).

RDF constructs were electroporated into *E. coli* BL21(DE3), and TerL constructs were electroporated into Origami (DE3). Origami cells have an oxidizing intracellular environment, which we found slightly increased the amount of visible precursor compared to a nonoxidizing strain for the TerL constructs. Overnight cultures were subcultured 1:100 into fresh LB medium and grown at 37°C with aeration to mid-log phase (optical density at 600 nm [OD_600_] of ~0.5). Cells were then induced with 0.5 mM IPTG (isopropyl-β-d-thiogalactopyranoside) for 1 h at 30°C for RDF constructs or 37°C for TerL constructs. Splicing assays and visualization of GFP-containing products were then performed (35).

For cleavage assays, two mycobacteriophage pairs were examined, BAKA (intein-plus) with Courthouse (intein-minus) and Bethlehem (intein-plus) with Solon (intein-minus). DNA substrate was produced from phage lysate by PCR (see Table S5 in the supplemental material). DNA was purified using the QIAquick PCR purification kit (Qiagen) and eluted in cleavage buffer (10 mM Tris, pH 8.0, 10 mM MgCl_2_, 25 mM KCl)*.* Overnight cultures of MG1655 (DE3) containing MIG constructs were subcultured as described above and induced for 2 h at 30°C with 0.5 mM IPTG. Protein expression was stopped with spectinomycin (100 µg/ml). Cells were lysed by sonication, and crude MIG lysate was used as the source of intein endonuclease. Lysate was diluted 1/25 in cleavage buffer with 1 µg of substrate DNA per reaction. Reactions were carried out in cleavage buffer, reaction mixtures were incubated for 30 min at 37°C, and then reactions were stopped. As controls, lysate from MIG TerL1-b (BAKA) was mixed with Solon TerL DNA substrate and lysate from MIG TerL1-c (Bethlehem) was mixed with Courthouse TerL DNA (Fig. 3C). Cleavage was visualized on a 1% agarose gel using EZ-Vision DNA dye (Amresco).

## SUPPLEMENTAL MATERIAL

Figure S1  Full terminase structure models. The full Phyre2 structure models of Minerva gp9 (terminase_1), Chandler gp6 (terminase_6), and ScottMcG gp245 (Pham3880) are shown. The intein insertion sites are represented by the +1 residues (red spheres), and the P-loop (cyan), Walker B (green), and C-motif (blue) are colored. The N and C termini are indicated. Models were manipulated in PyMOL (v1.7.2). Download Figure S1, EPS file, 1 MB

Figure S2  Expansion of horizontal gene transfer analysis. (A) Amino acid sequence identity between mycobacteriophage and actinobacterial inteins. A few cases of unexpectedly high similarity were detected in pairwise comparisons of mycobacteriophage and actinobacterial inteins, implying horizontal transfer (red squares), including (1) TdS inteins of mycobacteriophages and *Streptomyces*, (2) RecB and TerL1-d inteins in mycobacteriophages, and (3) mycobacteriophage TerL1-a and *K. rhizophila* TerL inteins. The amino acid sequence identity in other comparisons was ≤30%, e.g., (4) TerL1-e and *M. smegmatis* prophage TerL inteins, (5) mycobacteriophage Alice NT and SufB-b in *M. leprae* inteins, and (6) mycobacteriophage Alice NT and GyrA-a *Mycobacterium xenopi* inteins. (B and C) The relationships between class 1 (B) and class 3 (C) mycobacteriophage and mycobacterial inteins are shown. Unrooted ML trees are based on splicing block alignments of mycobacteriophage and mycobacterial class 1 or 3 inteins. Mycobacteriophages are in red, and mycobacteria are in black. Values of significant external nodes higher than 75% are shown, and the scales indicate the numbers of substitutions per site. Trees were made as described in the legend to Fig. 2D. Download Figure S2, EPS file, 0.2 MB

Figure S3  Alignment of TerL endonuclease domains. Endonuclease domains present in mycobacteriophages BAKA and Bethlehem. The sequence between splicing blocks B and F was aligned for BAKA gp6 (TerL1-b) and Bethlehem gp10 (TerL1-c) using MUSCLE and trimmed to show only conserved sequence blocks in the endonuclease domains. These inteins are used for cleavage assays (Fig. 3C). The alignment shows the presence of the four canonical LAGLIDADG endonuclease domains in blocks C, D, E, and H for BAKA and Bethlehem with the actual LAGLIDADG motifs in blocks C and E. Numbers indicate the residue position relative to the intein, and conservation is shown by shades of gray. Download Figure S3, EPS file, 0.2 MB

Figure S4  Pathways of class 1 and class 3 splicing. Class 1: step 1, the first cysteine (1; yellow) acts as a nucleophile and attacks the preceding amide bond (red arrow); step 2, a second attack by C+1; step 3, the terminal asparagine (green) cyclizes, releasing the intein; step 4, the ligated exteins form the native peptide bond. Class 3: step 1, the initial attack is performed by an internal cysteine in block F; step 2, the internal thioester is then attacked by C+1; steps 3 and 4 proceed as for class 1. Download Figure S4, EPS file, 0.2 MB

Figure S5  Alignment of the consensus splicing block sequence for class 1 and class 3 mycobacteriophage inteins. The class 3 WCT motif is shown in red lettering, and the percent identity at each position is shown by the shade of gray. Download Figure S5, EPS file, 0.4 MB

Table S1  All analyzed mycobacteriophages. Table S1, XLSX file, 0.1 MB

Table S2  All intein-containing mycobacteriophages and their inteins. Table S2, XLSX file, 0.04 MB

Table S3  Percent identity between the splicing domains of mycobacteriophage TerL1-c and -e and mycobacterial DnaB-b inteins. Table S3, PDF file, 0.01 MB

Table S4  Bacterial strains and constructs. Table S4, PDF file, 0.3 MB

Table S5  List of oligonucleotides used in present study. Table S5, PDF file, 0.02 MB
