# Supplementary material for: Mycobacteriophages as Incubators for Intein Dissemination and Evolution
Source: mBio. 2016 Oct 4;7(5):e01537-16. doi: 10.1128/mBio.01537-16 (PMC5050341; doi:10.1128/mBio.01537-16)
Supplement: Table S3 — Percent identity between the splicing domains of mycobacteriophage TerL1-c and -e and mycobacterial DnaB-b inteins. [file mbo005163016st3.pdf]

Table S3. Percent identity between the splicing domains of mycobacteriophage TerL1-**c** and -**e** and mycobacterial DnaB-**b** inteins

		TerL1-**c**				TerL1-**e**
		Anglerfish	Bethlehem	Petp2012	Violet	Gaia
DnaB-**b**	*M. asiaticum*	41.1%	45.3%	45.3%	44.2%	54.7%
	M. chubuense	44.2%	46.3%	46.3%	46.3%	54.7%
	M. fortiutum	42.1%	47.4%	47.4%	46.3%	54.7%
	M. leprae	41.1%	46.3%	46.3%	45.3%	52.6%
	M. lepromatosis	41.1%	46.3%	46.3%	45.3%	52.6%
	M. rhodesiae	46.3%	51.6%	51.6%	50.5%	52.6%
	M. septicum	44.2%	47.4%	47.4%	46.3%	54.7%
	M. smegmatis	42.1%	47.4%	47.4%	46.3%	54.7%
