# Supplementary material for: Mycobacteriophages as Incubators for Intein Dissemination and Evolution
Source: mBio. 2016 Oct 4;7(5):e01537-16. doi: 10.1128/mBio.01537-16 (PMC5050341; doi:10.1128/mBio.01537-16)
Supplement: Table S4 — Bacterial strains and constructs. [file mbo005163016st4.pdf]

Table S4. Bacterial strains and constructs

**Strain/Plasmid**	**Features and Comments**	**Source**
***E. coli* Strains**		
DH5α	F⁻ *endAI recAl hsdRl7* (rK$^-$ mK$^-$) *deoR supE44 thi-J gyrA96 relA*	Gibco-BRL
MG1655 (DE3)	F⁻ (λDE3) *ilvG rfb50 rph*1	James Imlay
Origami (DE3)	Δ(*ara–leu*)*7697* Δl*acX74* Δ*phoA PvuII phoR araD139 ahpC galE galK rpsL* F'[*lac*$^+$ *lacI*) *pro*] *gor522*::Tn10 *trxB* (Kan^R , Str^R , Tet^R)	Novagen
BL21 (DE3)	F– *ompT hsd*S$_B$(r$_B^-$ m$_B^-$) *gal dcm* (λDE3)	Novagen
**Plasmids**		
pACYCDuet-1	Expression vector, T7 promoter, CamR	Novagen
pACYC MIG-SufB	Used as cloning backbone by removing *Mtu* SufB insert using ClaI/SphI	(35)
pACYC MIG-RDF WT	Bethlehem gp51 flanked by short extein sequences (N-Extein: IGAHEGNHD; C-Extein: SRARDYLSKN) cloned into ClaI/SphI site between MBP and GFP coding sequences in pACYCDuet-1 backbone. Has mutation in endonuclease domain (R157W)	Present Study
pACYC MIG-RDF G316H	Same as pACYC MIG-RDF WT with the native penultimate residue mutated to histidine (G316H)	Present Study
pACYC MIG-TerL1-**b**	BAKA gp6 intein flanked by short extein sequences (N-Extein: VSRQNGKGDL; C-Extein: SILEAIELAG) cloned into ClaI/SphI site between MBP and GFP coding sequences in pACYCDuet-1 backbone	Present Study
pACYC MIG-TerL1-**c**	Bethlehem gp10 intein flanked by short extein sequences (N-Extein: TIAAVSQDQ ; C-Extein: TKNTFSL) cloned into ClaI/SphI site between MBP and GFP coding sequences in pACYCDuet-1 backbone	Present Study
pACYC MIG-TerL1-**e**	Gaia gp2 intein flanked by short extein sequences (N-Extein: ASFIILNE; C-Extein: SHHMTATNGG) cloned into ClaI/SphI site between MBP and GFP coding sequences in pACYCDuet-1 backbone	Present Study
pACYC MIG-TerL6-**f**	Chandler gp6 intein flanked by short extein sequences (N-Extein: EASMILIQ; C-Extein: TRWHPEDLSG) cloned into ClaI/SphI site between MBP and GFP coding sequences in pACYCDuet-1 backbone	Present Study
pACYC MIG-Pham3880-**g**	ScottMcG gp245 intein flanked by short extein sequences (N-Extein: AMTYLASSPY; C-Extein: SKIGKFYEL) cloned into ClaI/SphI site between MBP and GFP coding sequences in pACYCDuet-1 backbone	Present Study
