# Supplementary material for: Mycobacteriophages as Incubators for Intein Dissemination and Evolution
Source: mBio. 2016 Oct 4;7(5):e01537-16. doi: 10.1128/mBio.01537-16 (PMC5050341; doi:10.1128/mBio.01537-16)
Supplement: Table S5 — List of oligonucleotides used in present study. [file mbo005163016st5.pdf]

Table S5. Oligonucleotides

**Oligo ID**	**Sequence (5' to 3')**	**Application**
IDT4787	tcgagggaaggccttacatgcatgctatcggggctcatgaaggg	Bethlehem gp51 intein amplification for MIG-RDF WT with SphI InFusion ends
IDT4788	cttctcctttgctcatatcgatgttcttggagagatagtcc	Bethlehem gp51 intein amplification for MIG-RDF WT with ClaI InFusion ends
IDT4789	caagatccacctgacccataactcccgggccagg	Mutagenesis of MIG-RDF G316 to H
IDT4790	ccctggcccgggagttatgggtcaggtggatcttg	Mutagenesis of MIG-RDF G316 to H
IDT4335	tcgagggaaggccttacatgcatgctgttagccggcagaacggc	BAKA gp6 intein amplification for MIG-TerL1-**b** with SphI InFusion ends
IDT4336	cttctcctttgctcatatcgatcccagcgagctcaatggc	BAKA gp6 intein amplification for MIG-TerL1-**b** with ClaI InFusion ends
IDT4337	tcgagggaaggccttacatgcatgctacgatcgccgcggtctcc	Bethlehem gp10 intein amplification for MIG-TerL1-**c** with SphI InFusion ends
IDT4338	cttctcctttgctcatatcgatcagcgagaacgtgttcttcgt	Bethlehem gp10 intein amplification for MIG-TerL1-**c** with ClaI InFusion ends
IDT4341	tcgagggaaggccttacatgcatgctgccagttttattatcttgaatg	Gaia gp2 intein amplification for MIG-TerL1-**e** with SphI InFusion ends
IDT4342	cttctcctttgctcatatcgatcccaccattagtggcggt	Gaia gp2 intein amplification for MIG-TerL1-**e** with ClaI InFusion ends
IDT4345	tcgagggaaggccttacatgcatgctgaggcgtccatgatcctg	Chandler gp6 intein amplification for MIG-TerL6-**f** with SphI InFusion ends
IDT4346	cttctcctttgctcatatcgatgccggacaggtcttcggg	Chandler gp6 intein amplification for MIG-TerL6-**f** with ClaI InFusion ends
IDT4343	tcgagggaaggccttacatgcatgctgccatgacttacctggca	ScottMcG gp245 intein amplification for MIG-Pham3880-**g** with SphI InFusion ends
IDT4344	cttctcctttgctcatatcgatgagctcgtagaacttgcc	ScottMcG gp245 intein amplification for MIG-Pham3880-**g** with ClaI InFusion ends
IDT4460	gatcggtaccgtggcggttcactacccg	DNA substrate amplification of Solon *terL* ORF for cleavage assay

IDT4461	gatcaagctttcatcgcagcagacccg	DNA substrate amplification of Solon *terL* ORF for cleavage assay
IDT4558	gagcaccacggtgataaaca	DNA substrate amplification of upstream Courthouse *terL* ORF for cleavage assay
IDT4561	acaatgggctgaatgtcgat	DNA substrate amplification of upstream Courthouse *terL* ORF for cleavage assay
